# Factors associated with cystic fibrosis mortality before the age of 30: retrospective analysis of a cohort in southern Brazil

**DOI:** 10.1590/1414-431X2024e13476

**Published:** 2024-08-23

**Authors:** J. De Conto, P.T.R. Dalcin, B. Ziegler

**Affiliations:** 1 Programa de Pós-Graduação em Ciências Pneumológicas, Universidade Federal do Rio Grande do Sul, Porto Alegre, RS, Brasil; 2 Hospital de Clínicas de Porto Alegre, Porto Alegre, RS, Brasil

**Keywords:** Aging, Cystic fibrosis, Longevity, Survival

## Abstract

The aim of this study was to retrospectively evaluate the factors associated with mortality before the age of 30 in adults with cystic fibrosis (CF) followed up at a referral center in southern Brazil. This study included individuals over 18 years of age. Clinical data related to childhood and the period of transition to an adult healthcare of individuals with CF were recorded, as well as spirometric and mortality data of individuals between 18 and 30 years of age. A total of 48 patients were included in this study, of which 28 (58.3%) were male. Comparing groups, we observed a higher prevalence of homozygosis for the F508del mutation (P=0.028), massive hemoptysis before the age of 18 (P=0.027), and lower values of pulmonary function, forced expiratory volume in the first second (FEV1) (%) (P=0.002), forced vital capacity (FVC) (%) (P=0.01), and FEV1/FVC (%) (P=0.001) in the group that died before age 30. F508del homozygosis, episodes of massive hemoptysis in childhood, and lower FEV1 values at age 18 were related to mortality before age 30 in a cohort of individuals with CF in southern Brazil.

## Introduction

Cystic fibrosis (CF) is a genetic autosomal recessive disorder that causes a mutation in CF transmembrane conductance regulator (CFTR) protein, resulting in abnormalities in epithelial ionic transport (1,2). This change in chlorine channels leads to increased viscosity of body secretions that can lead to obstruction, inflammation, and destruction of organs, mainly the lungs and pancreas (3).

After the initial description of CF in 1938, several studies were conducted bringing scientific and technological advances, from the standardization of the sweat test in 1958, to the development of CFTR-modulating drugs (2,4,5) today. Data from the 2020 North American CF registry show that the predicted survival median was age 50 years (95% confidence interval; 48.5-51.3 years) (6). Thus, the population of individuals aged 18 or older has progressively increased (4).

According to data from the Brazilian Cystic Fibrosis Registry (REBRAFC), the records of individuals with CF in Brazil has been increasing in recent years, with about 25% of them being older than 18 years (7). With the growth and aging of this population, the prevalence of age-related diseases and disease progression increased, changing the health needs of these patients (1,4,8).

Several studies have shown that the survival of individuals with CF is directly related to the initial presentation of the disorder, considering that patients with early onset of respiratory symptoms have a shorter survival (9). Other relevant factors that lead to lower survival are related to age at diagnosis (mainly in countries where neonatal screening is not available), presentation of pneumothorax, pulmonary colonization by *Pseudomonas aeruginosa* before starting in adult healthcare, use of intravenous and aerosol antibiotics during childhood and adolescence, as well as low socioeconomic status (9-12).

However, few studies provide data regarding survival in Brazil. Therefore, it is relevant to verify the proportion of patients with CF that evolve to death before the age of 30 years and identify which clinical characteristics influence their survival. Understanding these variables can help the multidisciplinary team to adapt clinical procedures, improve referral criteria for lung transplantation, and ultimately achieve greater longevity in this population.

This study aimed to retrospectively evaluate the factors associated with mortality between 18 and 30 years in adult individuals with CF followed up at a referral center in southern Brazil.

## Material and Methods

### Study setting and participants

This was a retrospective cohort study of patients with CF born in 1990 or earlier, diagnosed before age 18, and assisted by the Adults CF Program at the Hospital de Clínicas de Porto Alegre (HCPA), with evaluation of outcomes until December 31, 2020 (inclusion criteria).

Exclusion criteria were late or inconclusive diagnoses, patients with incomplete medical records, and those who had other causes of death unrelated to CF. Patients who started late follow-up in the adult healthcare and with no data available at the time of transition to the adult healthcare were also excluded.

In this study, the sample was non-probabilistic and intentional, which included all patients assisted by the Program for Teenagers and Adults with CF at HCPA who met the inclusion and exclusion criteria.

### Proceedings

Individuals were identified using the Adults CF Program database. After verifying the inclusion and exclusion criteria, a data collection form was filled out for later elaboration of a database in the Microsoft Office^®^ Excel 2020 program (USA).

Age 18 was chosen as the study entry date because that is when the transition to the Adults CF Program at HCPA formally occurs. Therefore, a search was performed to find sociodemographic data, gene mutation, records of CF-related complications during childhood, such as pneumothorax, massive hemoptysis, allergic bronchopulmonary aspergillosis, bacterial lung infection, records of liver disease, pancreatic insufficiency, diabetes related to CF, hospital admissions, need for admission to an intensive care unit (ICU), use of intravenous and aerosolized antibiotic therapy, history of liver or lung transplantation, use of home oxygen therapy, pulmonary function test, and body mass index (BMI), related to the moment of transition of each patient.

Genotyping was carried out at one central laboratory - Mendelics Laboratory, Brazil. Genomic DNA was extracted from saliva samples using a standard protocol with a QiaSymphony Midi Kit (Qiagen, Germany). Genotyping was performed using the Genome Analysis Toolkit (GATK), version 4.1.4.1 (https://gatk.broadinstitute.org/).

Massive hemoptysis was characterized by blood loss in a volume greater than 240 mL in 24 h, or more than 100 mL per day for several days (13,14). Pancreatic insufficiency was defined using pancreatic enzymes. CF-related diabetes mellitus (CFRDM) was identified using insulin.

Spirometry was performed by the Pulmonary Physiology Unit of the Pulmonology Service of the HCPA using the Jaeger equipment (v 4.31a; Jaeger, Germany). Pulmonary function parameters of forced expiratory volume in one second (FEV1) and forced vital capacity (FVC), expressed in liters (L), obtained at age 18 were recorded. Predicted percentage for age, sex, and height were estimated according to the equation by Pereira et al. (15).

The end date of the study for the survivors was December 31, 2020. The remaining patients were censored on the date of lung transplantation if this occurred before age 30, or on the date of their death.

### Statistical analysis

The collected data were inserted in the Microsoft Office Excel program (USA). First, using the Statistical Package for the Social Sciences (SPSS, IBM, USA), version 18.0, a descriptive analysis of the study variables was performed, followed by a normality analysis using the Kolmogorov-Smirnov and Shapiro-Wilk tests.

Qualitative data are reported as n (% of all cases), whereas quantitative data are reported as means±SD or as median and interquartile range (IQR). In the comparative analysis between death before age 30 and survivor groups, the Student's *t*-test was used for independent samples with continuous variables and normal distribution, and the Mann-Whitney U test was used for continuous variables without normal distribution. Fisher's exact test was used to analyze qualitative variables.

ROC curve and Kaplan-Meier analysis were performed in R 4.1.2 software program (https://cran.r-project.org/) using pROC and survival packages, respectively. In the ROC curve, the best cut-off point for FEV1 (%) at age 18 was obtained using the Youden criterion. For all analyses, P-values <0.05 were considered statistically significant.

### Ethical aspects

The project, which followed resolution No. 466/2012, was forwarded to the Research Ethics Committee of the HCPA, and after its approval, data collection began. All authors signed a confidentiality agreement for the use of the data, guaranteeing the confidentiality of individual’s data.

## Results

**Figure 1:**
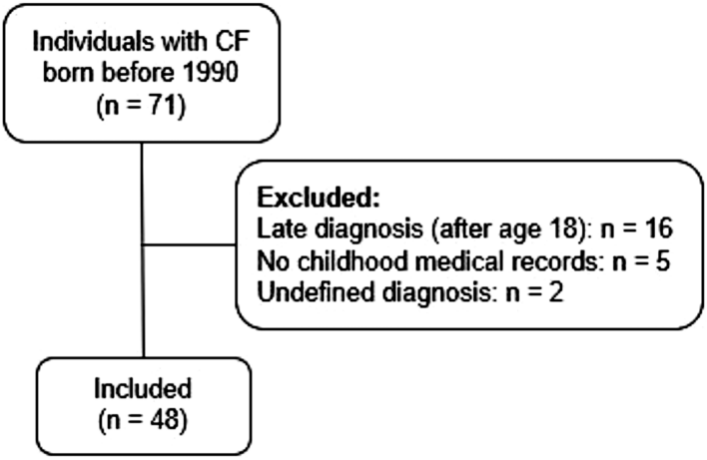
Flow diagram of selection of patients with cystic fibrosis (CF).

In the Adults CF Program database, 71 patients were born before 1990. A total of 23 patients were excluded from the sample; 16 due to late diagnosis (after age 18), five due to not having childhood data in the medical records, and two due to having an undefined diagnosis (Figure 1).

In the end, 48 patients with a median age of diagnosis of 1 year were included in this study, of which 28 (58.3%) were male. In the initial presentation of the disease, respiratory symptoms (87.5%), steatorrhea (62.5%), and family history (25%) were the main features. A total of ten patients (20.8%) were homozygous for the F508del mutation (Table 1).

**Table 1:** General characteristics of the sample (n= 48).

Characteristics	Data
Gender	
Male, n (%)	28 (58.3)
Female, n (%)	20 (41.7)
Age at diagnosis (years), median (IQR)	1.0 (2.0)
F508del homozygosity, n (%)	10 (20.8)
Family history, n (%)	12 (25)
CF related complications before 18 years	
Pancreatic insufficiency, n (%)	39 (81.3)
CFRDM, n (%)	3 (6.3)
Massive hemoptysis, n (%)	5 (10.4)
MSSA, n (%)	29 (60.4)
MRSA, n (%)	9 (18.8)
*Pseudomonas aeruginosa*, n (%)	41 (85.4)
*Burkholderia cepacia*, n (%)	13 (27.1)
Hospital admission, n (%)	40 (83.3)
ICU admission, n (%)	4 (8.3)
Invasive mechanical ventilation, n (%)	5 (10.4)
Clinical data at age 18 years	
Body mass index (kg/m^2^), mean (SD)	20.4 (2.3)
FEV1 (%), mean (SD)	64 (22.2)
FVC (%), mean (SD)	73.8 (18.5)
FEV1/FVC (%), mean (SD)	87 (12.4)
Death, n (%)	16 (33.3)
Death <30 years, n (%)	8 (16.7)
Age at death, mean (SD)	31.3 (5.6)

Data are reported as n (%), median (interquartile range), or mean (SD). CF: cystic fibrosis; CFRDM: CF related diabetes mellitus; MSSA: methicillin-sensitive *Staphylococcus aureus*; MRSA: methicillin*-*resistant *Staphylococcus aureus*; ICU: intensive care unit; FEV1: first second of forced expiratory volume; FVC: forced vital capacity.

When comparing groups, the group of patients who died before age 30 had a significantly higher prevalence of homozygosis for the F508del mutation (P=0.028), massive hemoptysis before age 18 (P=0.027), and lower values of lung function [FEV1 (%) (P=0.002), FVC (%) (P=0.01), FEV1/FVC (%) (P=0.001)] in relation to survivors (Table 2). Other characteristics analyzed, like gender, early respiratory symptoms, and pathogen colonization, did not demonstrate statistically significant differences (Table 2).

**Table 2:** General characteristics of cystic fibrosis (CF) patients according to 30-year mortality.

Characteristics	Death <30 years(n=8)	Survivors(n=40)	P
Male, n (%)	6 (75)	22 (55)	0.440
Initial presentation of the disease			
Diagnostic age (years), median (IQR)	1.5 (6.25)	2.5 (9.75)	0.559
Respiratory symptoms, n (%)	7 (87.5)	35 (87.5)	1.0
Steatorrhea, n (%)	7 (87.5)	23 (57.5)	0.229
Meconium ileus, n (%)	0 (0)	3 (7.5)	1.0
F508del homozygosity, n (%)	4 (50)	6 (15)	0.028*
CF-related complications before age 18			
Pancreatic insufficiency, n (%)	7 (87.5)	32 (80)	1.0
CFRDM, n (%)	1 (12.5)	2 (5)	0.429
Use of ursodeoxycholic acid, n (%)	4 (50)	15 (37.5)	0.695
Massive hemoptysis, n (%)	3 (37.5)	2 (5)	0.027*
MSSA, n (%)	4 (50)	25 (62.5)	0.695
MRSA, n (%)	2 (25)	7 (17.5)	0.633
*Pseudomonas aeruginosa*, n (%)	8 (100)	33 (82.5)	0.583
*Burkholderia cepacia*, n (%)	3 (37.5)	10 (25)	0.664
ABPA, n (%)	2 (25)	1 (2.5)	0.068
Intravenous azithromycin, n (%)	7 (87.5)	24 (60)	0.230
Inhaled colistin, n (%)	4 (50)	16 (40)	0.703
Inhaled tobramycin, n (%)	4 (50)	12 (30)	0.413
Hospital admission, n (%)	8 (100)	32 (80)	0.320
ICU admission, n (%)	1 (12.5)	3 (7.5)	0.530
Invasive mechanical ventilation, n (%)	2 (25)	3 (7.5)	0.189
Clinical data at age 18 years			
Body mass index (kg/m^2^), mean (SD)	19.99 (2.20)	20.42 (2.37)	0.640
FEV1 (L), mean (SD)	1.85 (0.54)	2.73 (0.88)	0.01*
FEV1 (%), mean (SD)	42.5 (10.73)	68.7 (21.3)	0.002*
FVC (L), mean (SD)	2.87 (0.81)	3.51 (0.95)	0.088
FVC (%), mean (SD)	58.87 (12.35)	77 (18.15)	0.01*
FEV1/FVC (absolute), mean (SD)	64.6 (6.4)	77.3 (9.81)	0.001*
FEV1/FVC (%), mean (SD)	74.75 (7.59)	89.64 (11.69)	0.001*

Data are reported as n (%), median (interquartile range), or mean (SD). CF: cystic fibrosis; CFRDM: CF related diabetes mellitus; MSSA: methicillin-sensitive *Staphylococcus aureus*; MRSA: methicillin*-*resistant *Staphylococcus aureus*; ABPA: allergic bronchopulmonary aspergillosis; ICU: intensive care unit; FEV1: first second of forced expiratory volume; FVC: forced vital capacity; L: value in liters. *P<0.05.

The ability of FEV1 at age 18 to predict mortality at age 30 had an area under the ROC curve of 0.875. According to Youden's criterion, the best cut-off point was predicted FEV1 <59.5%, with a 67.6% specificity and 100% sensitivity.

**Figure 2:**
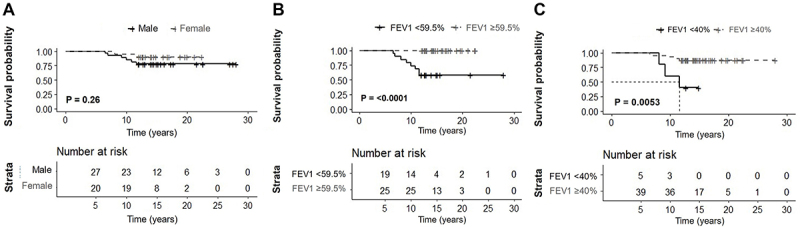
Kaplan-Meier curve of mortality according to sex (**A**) and stratified by forced expiratory volume in the first second (FEV1) considering the cutoff points 59.5% (**B**) and 40% (**C**).

Figure 2 shows the survival curves stratified by sex (Figure 2A) and FEV1. We observed that FEV1 <59.5% at age 18 was associated with a higher risk of mortality before age 30 (P=<0.0001) (Figure 2B). The same occurred for FEV1 <40% at age 18 (P=0.0053); in this situation, we estimated a median of survival of 12 years (Figure 2C).

## Discussion

This study retrospectively evaluated the factors associated with mortality before age 30 in adults with CF. A higher prevalence of homozygosis for the F508del mutation, a massive hemoptysis during childhood, and lower values of pulmonary function at age 18 were found in the group that died before age 30. FEV1 <59.5% at age 18 was associated with a higher risk of mortality before age 30.

The presence of the F508del mutation in homozygosis was significantly higher in the group that died before age 30. The class II CFTR variant, found in most individuals with CF, called F508del, is considered the most common disease-causing variant in southern Brazil (16,17). The defective protein is degraded before reaching the epithelial membrane and does not perform the transepithelial transport function, resulting in worse clinical outcomes, faster decline in lung function, and early mortality (2,16,18-20). This scenario has been changing in the last decade with the development of CFTR-modulating therapies. The use of triple therapy (elexacaftor, tezacaftor, and ivacaftor) in individuals who have one or two F508del variants has increased quality of life scores and increased predicted FEV1 by 14.3% compared to placebo controls (21). This therapy approach is recently being introduced to CF patients in Brazil (7).

Unlike our findings, other authors failed to find the F508del genetic alteration in homozygosis as a factor associated with mortality in individuals with CF over age 40 (22,23). Stephenson et al. (23) showed a similar increase in the survival of individuals with CF from 1990 to 2012 in Canada, regardless of their genotypic severity. Currently, the life expectancy of individuals with CF in the UK, regardless of their genetic mutation, is 50 years, both for those born today and for those who have reached 30 years or more (24). However, we must consider that most of the studies analyzing cohorts of adults with CF were conducted in reference centers in developed countries, which have a better organization of the health systems, disposing of neonatal screening, and lung transplant programs, that favor a better prognosis (4,22).

Massive hemoptysis is considered a common respiratory complication affecting approximately 20% of adults and approximately 4% of infants (25,26). This complication is associated with poor outcomes such as a greater number of ICU admissions, need for lung transplantation, and increased risk of mortality (14,27,28). In our study, 10% of the sample had hemoptysis in childhood, and this finding was more prevalent in the group that died before age 30.

To date, the impact of hemoptysis on the loss of lung function is unclear; however, studies have shown an association between the occurrence of episodes of massive hemoptysis and predicted FEV1 <40% (13,14). According to the Cystic Fibrosis Foundation, individuals with CF who have FEV1 <40% of predicted and episodes of massive hemoptysis requiring ICU admission should be referred to the lung transplant list (29). There are six lung transplantation centers in Brazil, distributed in 3 of the 27 Brazilian states (30). According to REBRAFC (7) in 2021, 47 individuals with CF underwent lung transplantation in Brazil.

Pulmonary function is a resource widely used to characterize the severity of lung disease in CF (31). Several studies have shown that the decrease in lung function, especially FEV1, is related to lower quality of life, higher morbidity, and early mortality, including children (1,31-34). Predicted FEV1 values <30% are related to a 40 to 50% chance of 2-year mortality (1,29). In our study, individuals with predicted FEV1 <59.5% at age 18 had a higher risk of mortality before age 30, as well as predicted FEV1 <40% at the time of transition to the adult healthcare, which had a median survival of 12 years.

In a previous study, Simmonds et al. (9) observed that higher values of FEV1 (%) at age 18 are associated with greater life expectancy of individuals with CF. It is already known that FEV1 (%) is the strongest predictor of mortality, and its predictive performance is improved when associated with other variables such as genotype, weight, height, and sputum microbiology (35,36). Our analysis did not show a statistically significant difference in other variables such as sex, age at diagnosis, BMI, and the presence of some pathogens in sputum during childhood, as observed in previous studies (9,23,37), which can be explained by the small sample size of this study.

The socioeconomic level of the sample was not assessed, a factor that could influence the longevity of individuals with CF (4,12,20). The retrospective design and the fact that it was conducted in a single reference center are other limitations of this study. The reference center used paper records until 2000, hampering data collection. We chose to analyze data related to diagnosis, comorbidities related to CF during childhood, and pulmonary function at age 18 aiming to assess associations with mortality at age 30; however, the time interval between entry into the study among some individuals was not considered in the sample, which may have influenced survival considering the advances in the clinical treatment of CF.

This is the first study that provides an analysis of survival and factors related to mortality in a Brazilian population with CF and long-term survival. Therefore, our analysis suggested that among the factors that influence the survival of individuals with CF at age 30 in southern Brazil are the presence of the F508del mutation in homozygosity, massive hemoptysis in childhood, and predicted FEV1 values <59.5% at age 18.
